# Supplementary material for: Evolution of Angiotensin Peptides and Peptidomimetics as Angiotensin II Receptor Type 2 (AT2) Receptor Agonists
Source: Biomolecules. 2020 Apr 23;10(4):649. doi: 10.3390/biom10040649 (PMC7226584; doi:10.3390/biom10040649)
Supplement: Supplementary file 1 [file biomolecules-10-00649-s001.pdf]

Supporting Information for

Evolution of Angiotensin Peptides and Peptidomimetics as AT2 Receptor Agonists

Silvana Vasile[1], Anders Hallberg[2], Jessica Sallander[1], Mathias Hallberg[3], Johan Åqvist[1] and Hugo Gutiérrez-de-Terán[1*]

[1] Department of Cell and Molecular Biology, Uppsala University, Biomedical Centre (BMC), BOX 596, SE-751 24 Uppsala, and Sweden and Science for Life Laboratory, BMC (H.G.T.); silvana.vasile@icm.uu.se (S.V.); johan.aqvist@icm.uu.se (J.Å.)
[2] Department of Medicinal Chemistry, Division of Organic Pharmaceutical Chemistry, BMC, Uppsala University, P.O. Box 574, SE-751 23 Uppsala, Sweden; anders.hallberg@ilk.uu.se
[3] The Beijer Laboratory, Department of Pharmaceutical Biosciences, Division of Biological Research on Drug Dependence, BMC, Uppsala University, P.O. Box 591, SE-751 24 Uppsala, Sweden; Mathias.Hallberg@farmbio.uu.se

* Correspondence: hugo.gutierrez@icm.uu.se; Tel.:+46(0)18-471-5056

Table of contents

Figure S1 2
Figure S2 3
Figure S3 4
Figure S4 5
Figure S5 6
Figure S6 7
Figure S7 8
Figure S8 9
Figure S9 9
Figure S10 10
Figure S11 10
Figure S12 11
Figure S13 12
Table S1 13

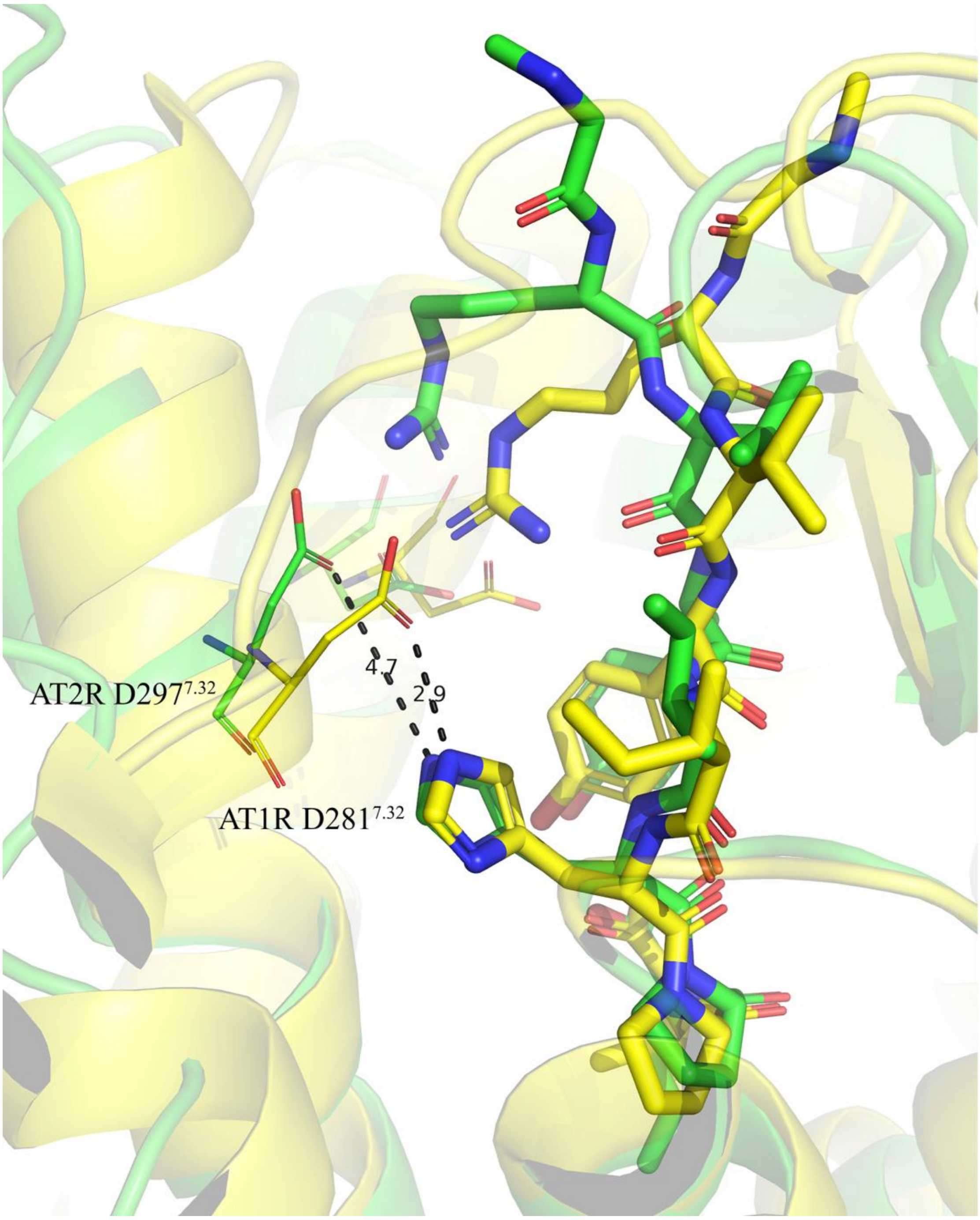

Figure S1 Comparison of the relative position of His⁶ in AT1R (yellow) and AT2R (green)

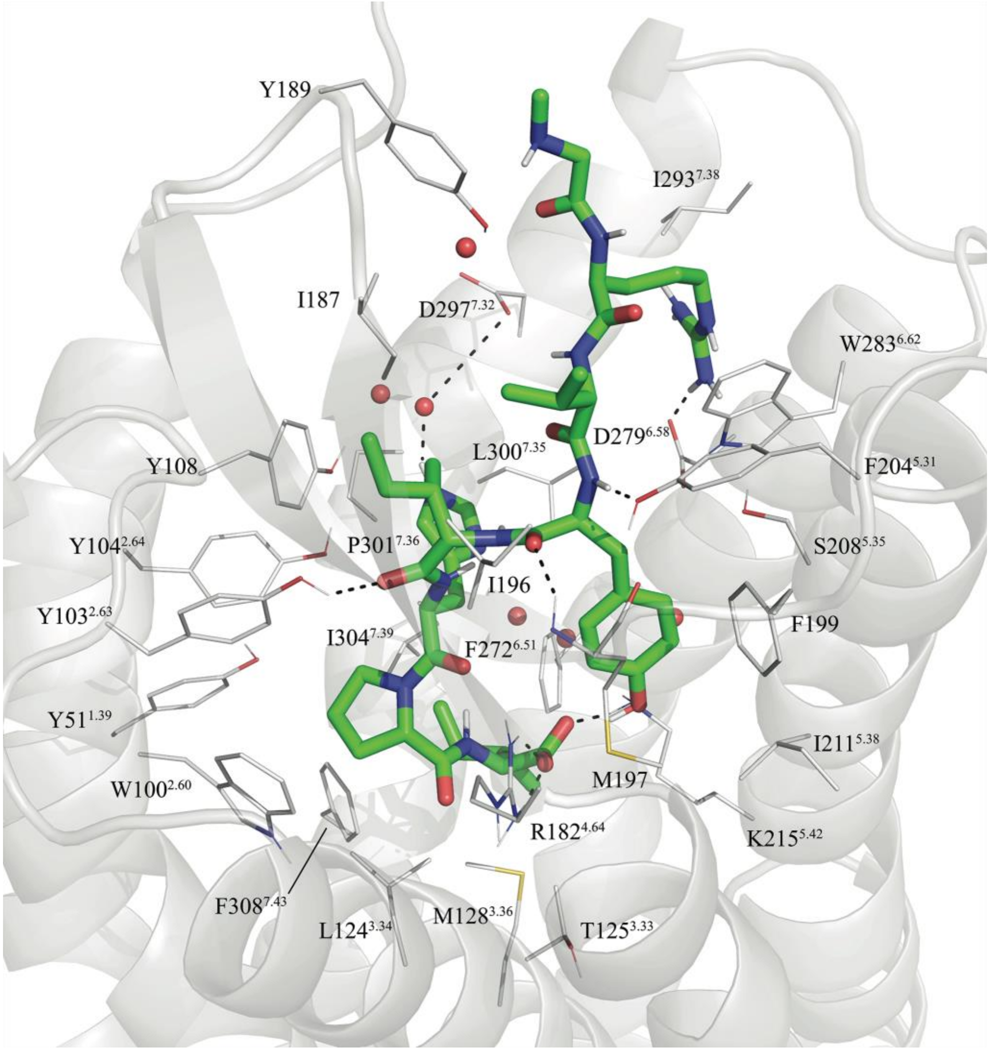

Figure S2: Binding pocket of sarile (**1**) in AT2R

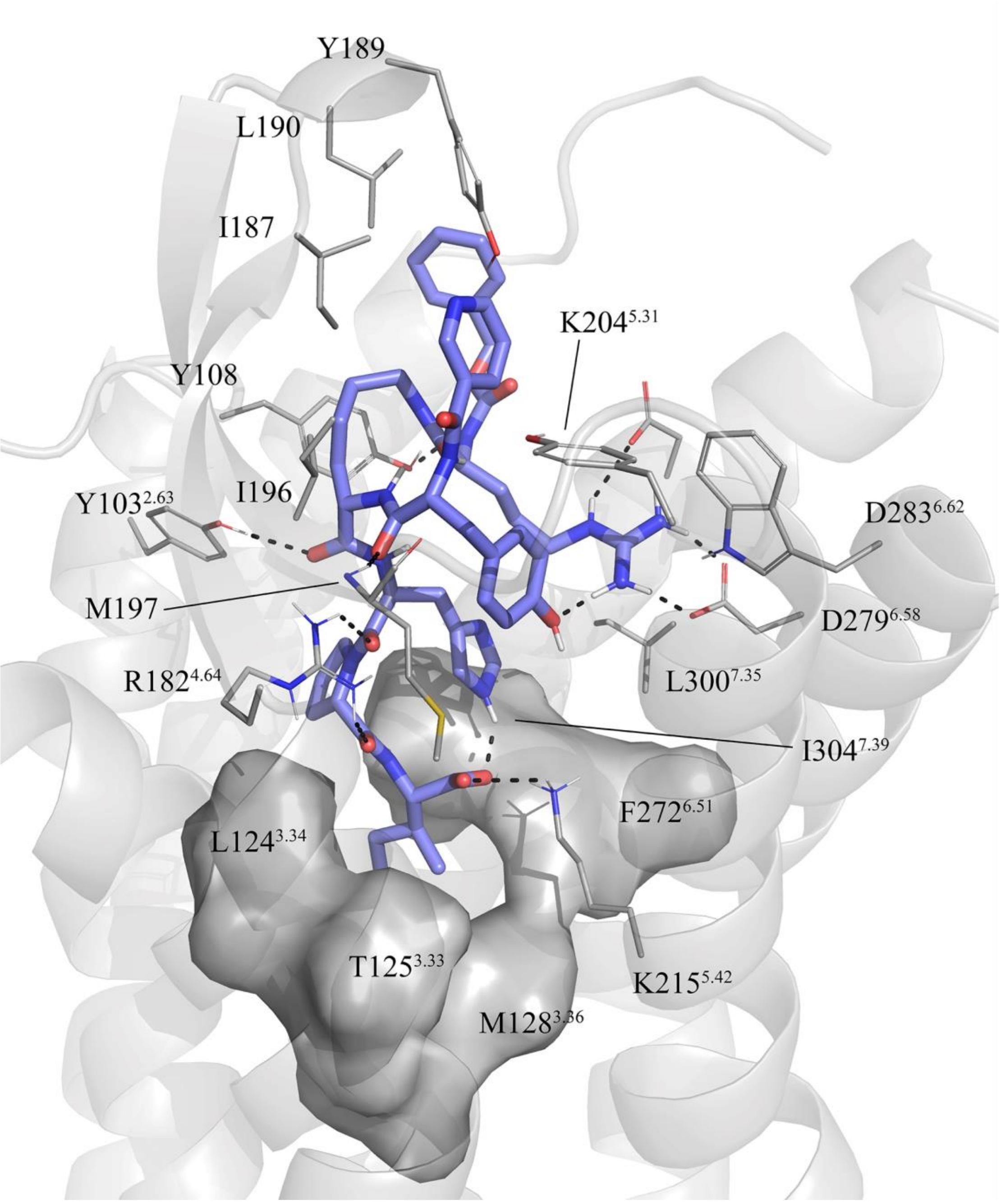

Figure S3: Binding mode of compound **2**

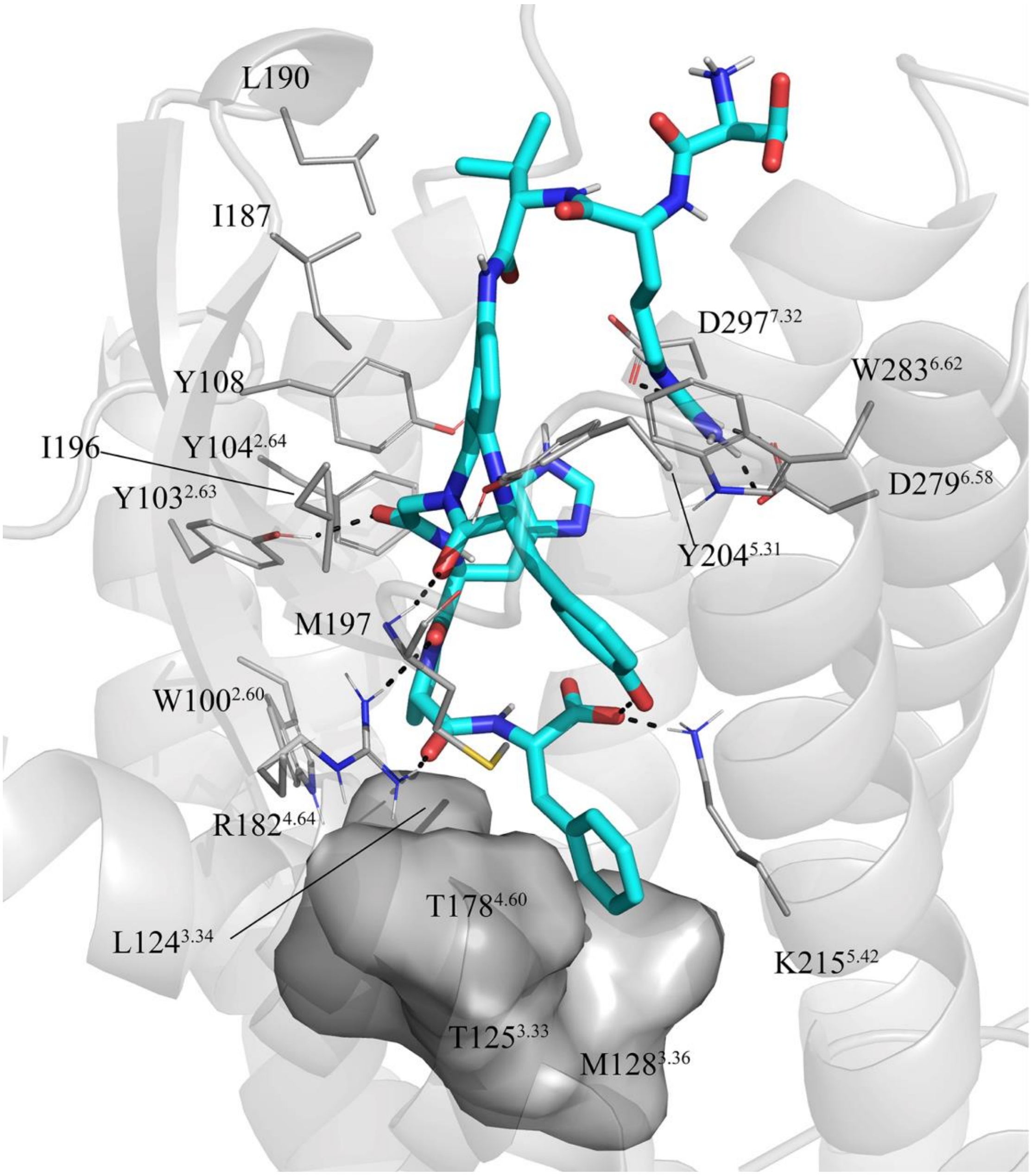

Figure S4: Binding mode of compound **3**

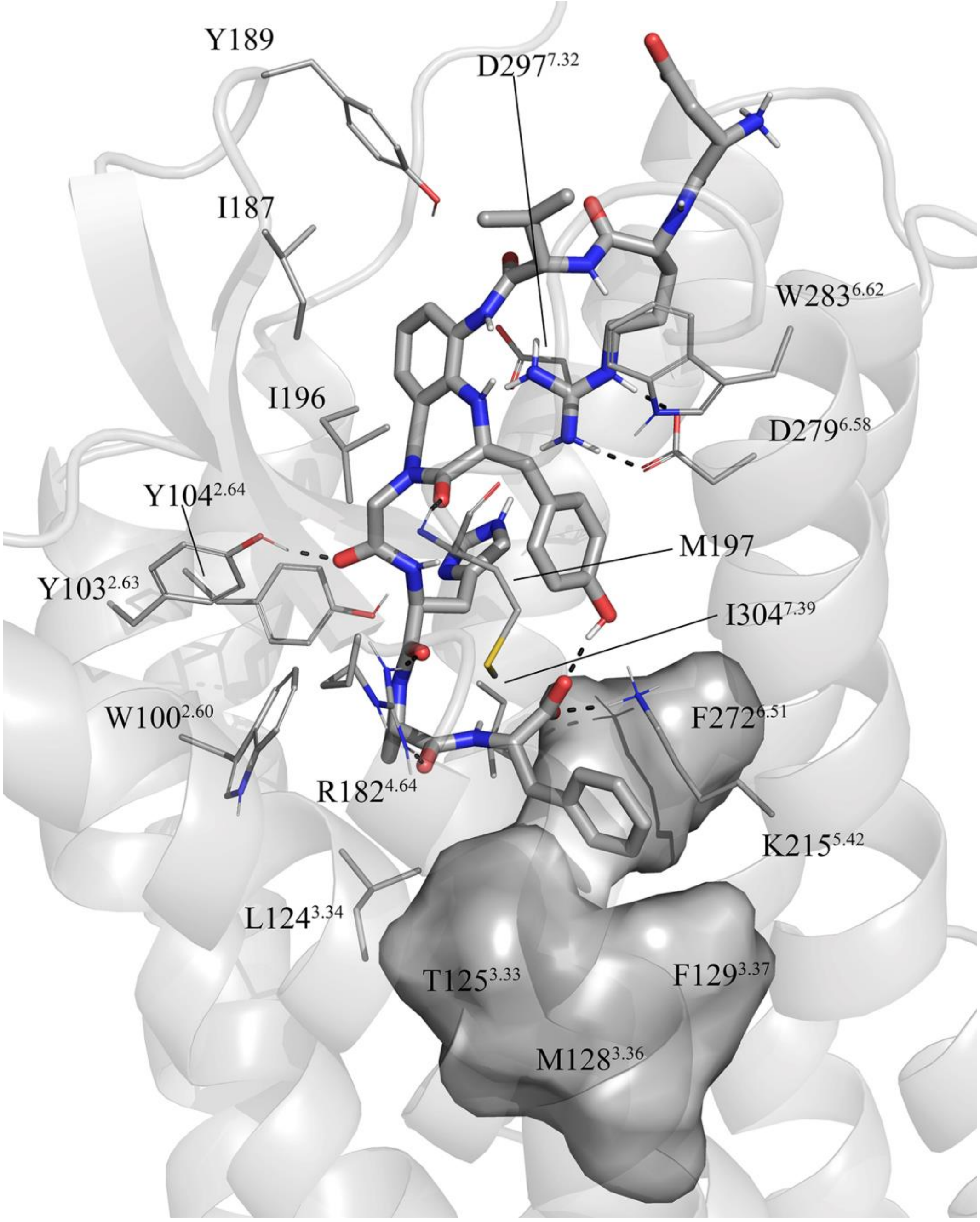

Figure S5: Binding mode of compound **4**

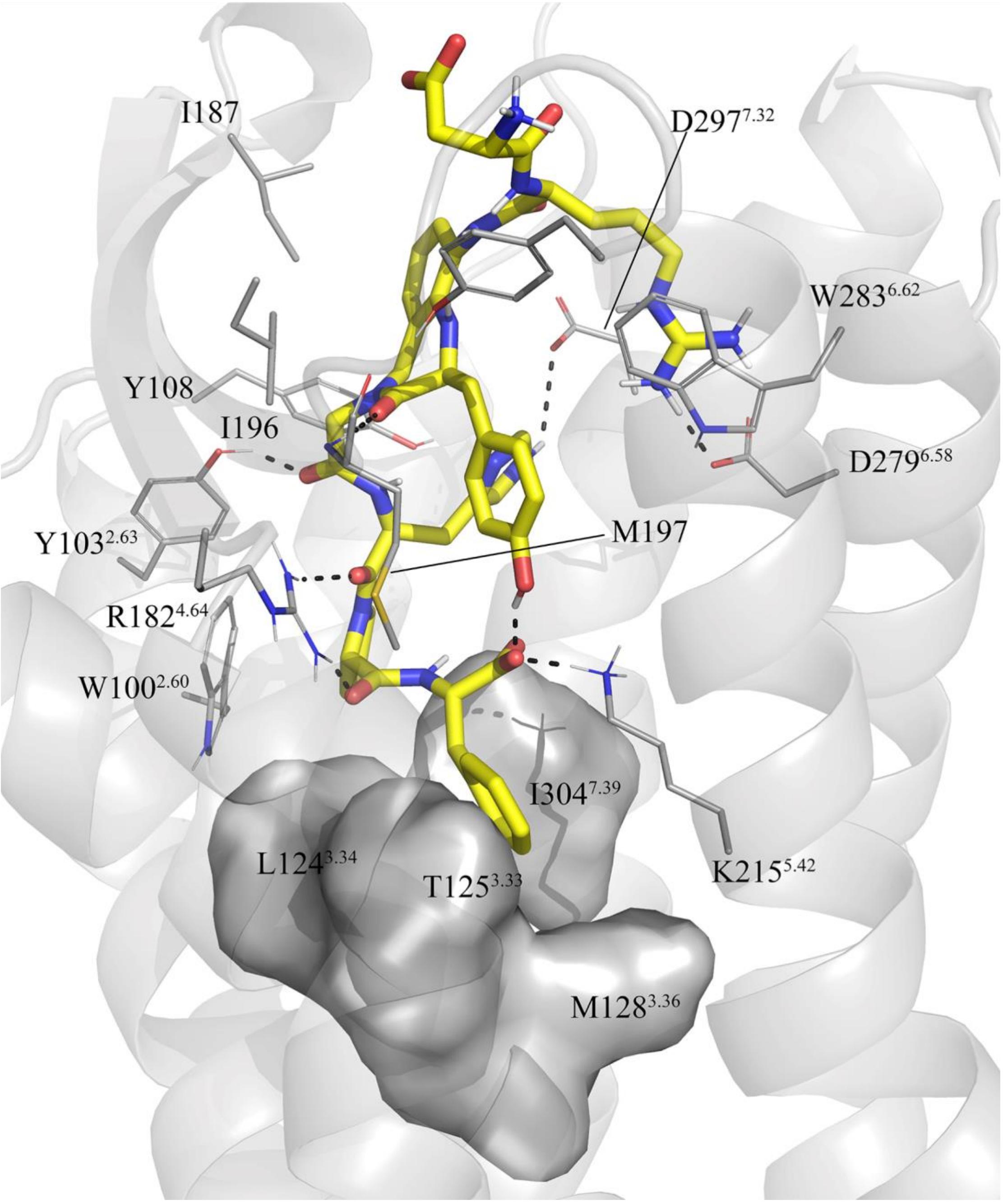

Figure S6: Binding mode of compound **5**

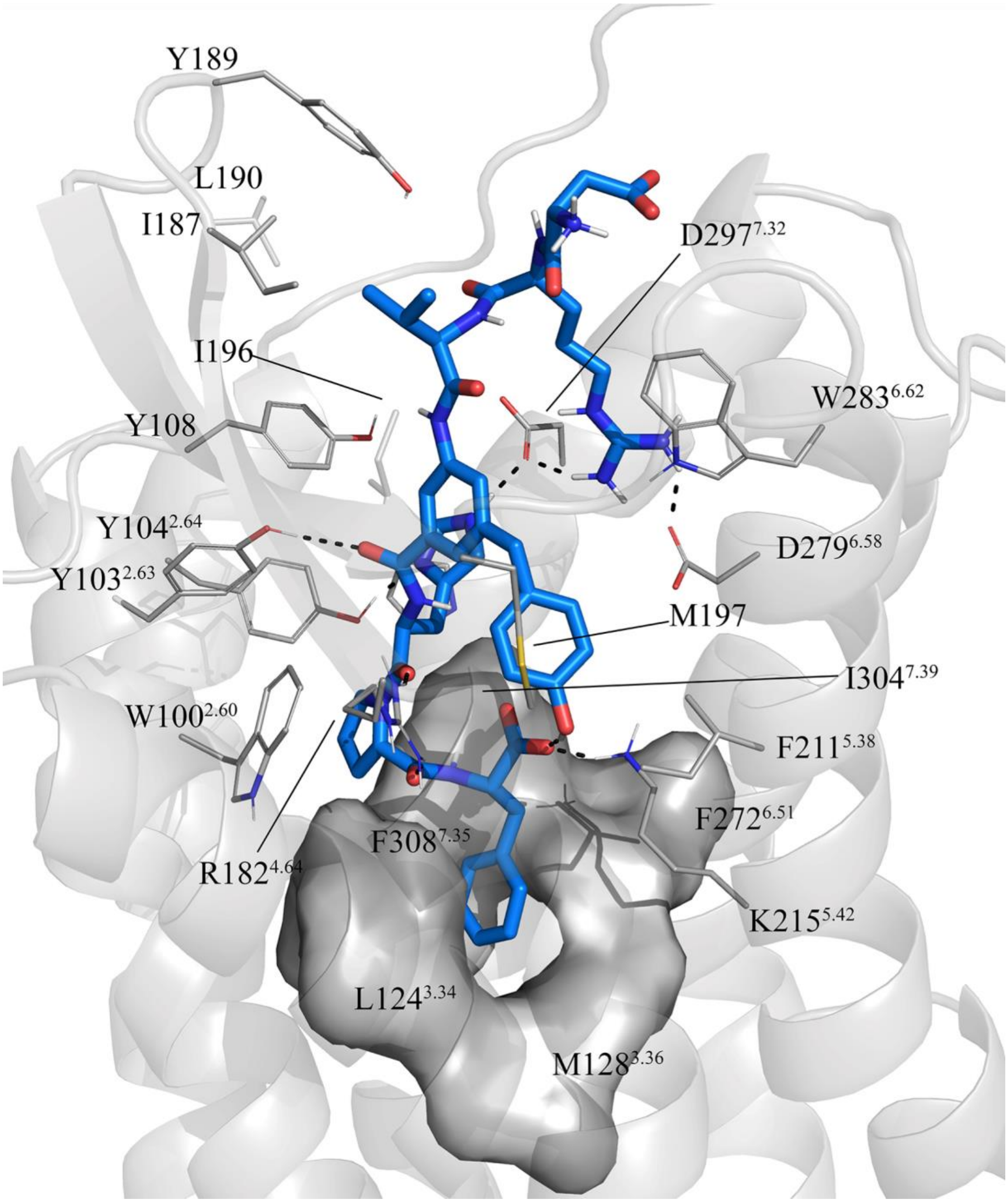

Figure S7: Binding mode of compound **6**

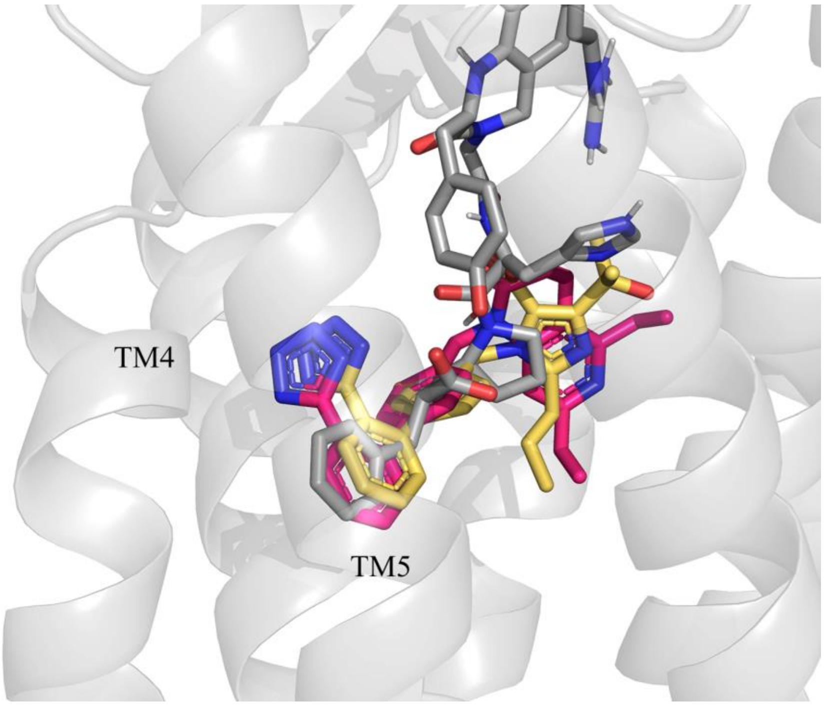

Figure S8: Comparison of the C-terminus orientation of **4** with co-crystallised AT2 antagonist

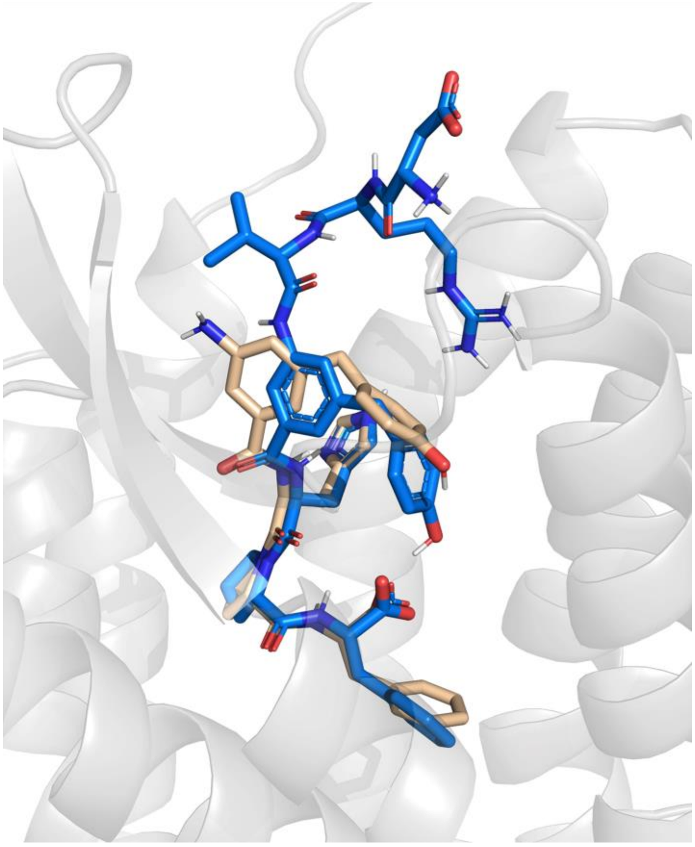

Figure S9: Comparison of the binding modes of **6** and **11**. The NH_2 of **11** is an extension of the gamma-turn mimic of **6**

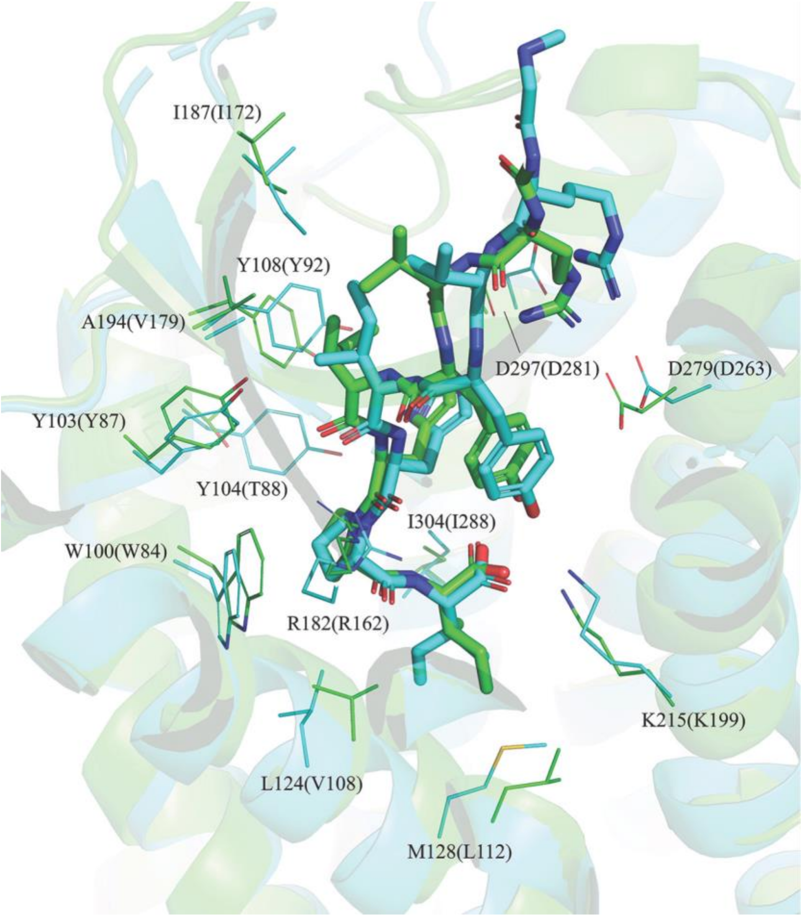

Figure S10: Comparison of the binding modes of sarile in AT1R (green) and AT2R (cyan).

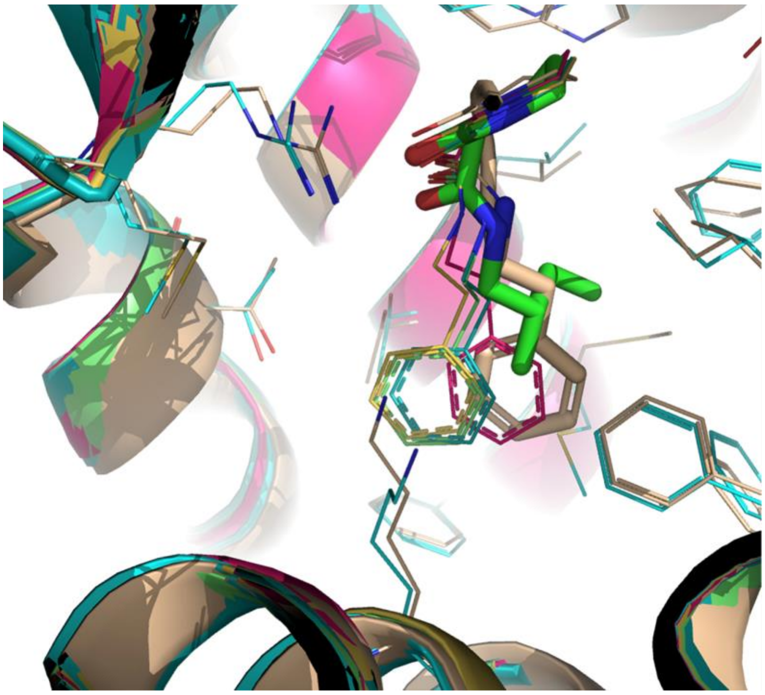

Figure S11: Sarile (green) and AngII (brown) C-terminal residues, overlaid with our docked compounds (green=3, cyan=4, yellow=5, magenta=6). The predicted rotamer of the Phe is actually the same as in AngII

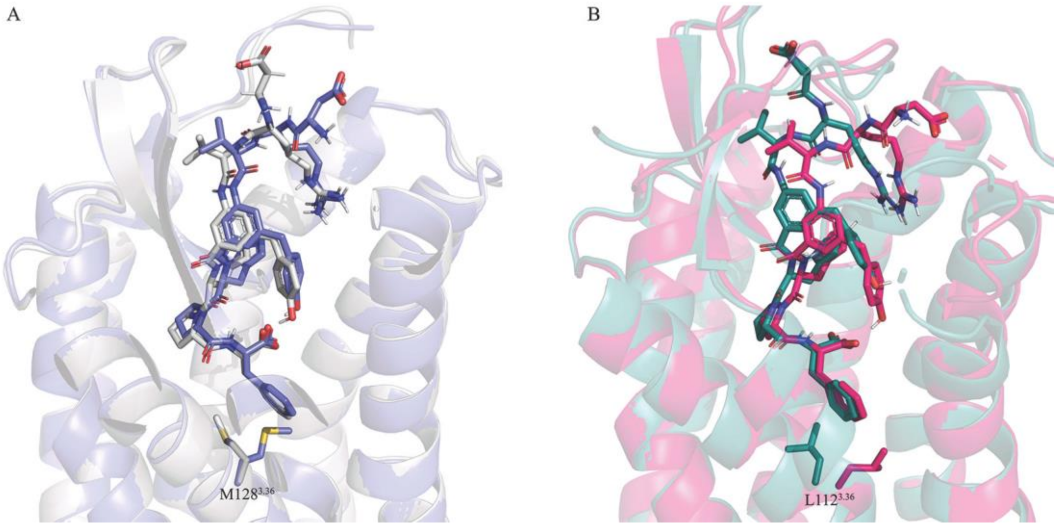

Figure S12: Comparison between the docking poses of compound 6 based on sarile or ATII-bound ATR structures. A) AT2R with sarile-based modelling in violet, AngII-based model in gray. B) AT1 structure, with sarile-based model in magenta, AngII-model in green.

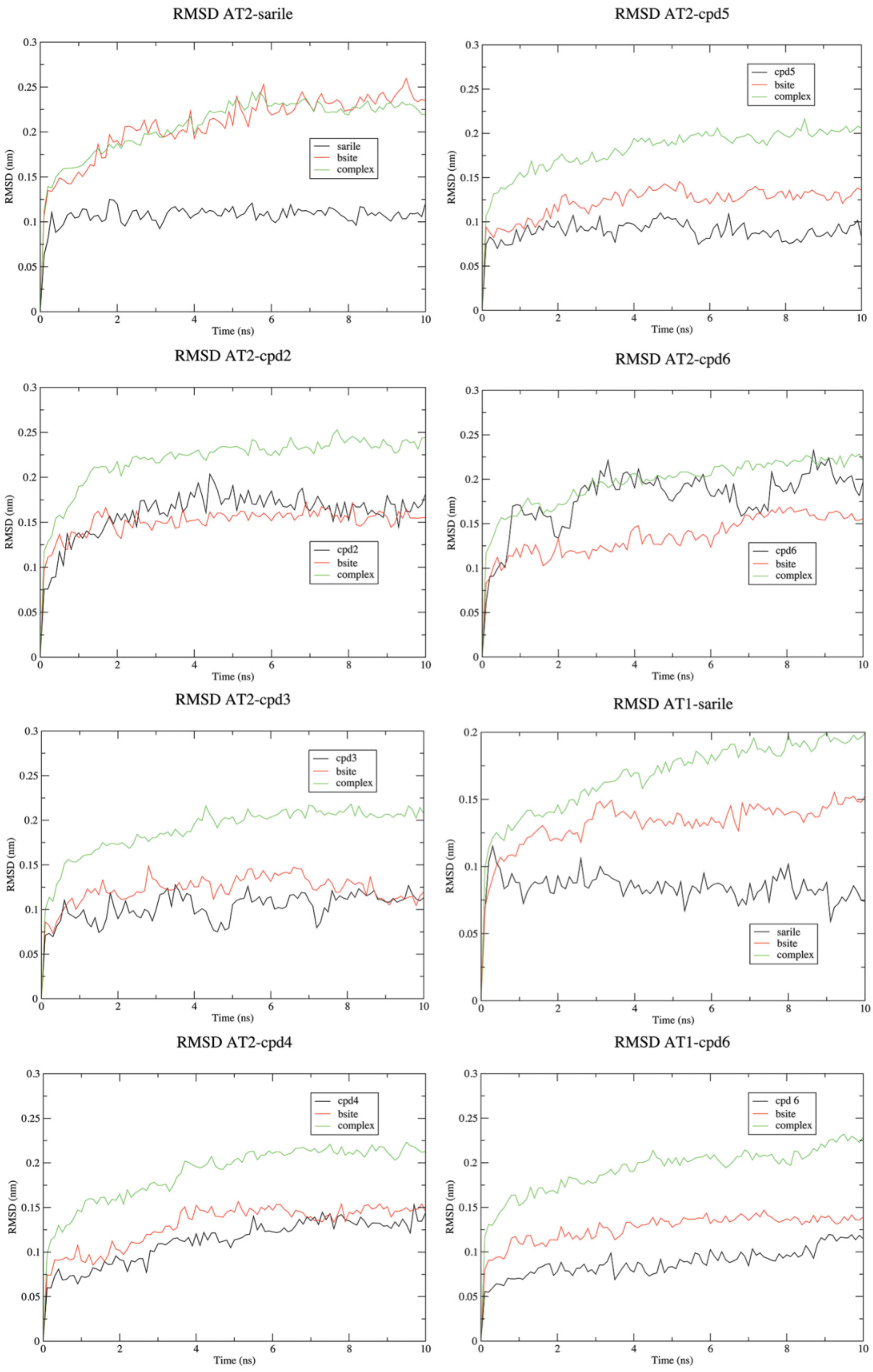

Figure S13: Average RMSD of each system subjected to 3 x 10 ns of MD simulations. Black lines represent the average RMSD of the ligands, red lines represent the average RMSD of the binding sites and green lines represent the average RMSD of the complexes (receptor-ligand).

Table S1: Relative binding affinity (in terms of experimental and calculated shifts in the free energy of binding) between two pairs of AT2 agonists, assuming the same binding pocket for the Phe/Ile sidechain on the C-terminus (related to Table 1).

**Mutation**	**Chemical modification**	**$\Delta\Delta G_{exp}$ ± s.e.m. (kcal/mol)**	**$\Delta\Delta G_{calc}$ ± s.e.m. (kcal/mol)**
**8 → $\mathbf{7_{alternative}}$**	Ile → Phe	1.54 ± 0.06	-0.41 ± 0.58
**10 → $\mathbf{9_{alternative}}$**	Ile → Phe	2.38 ± 0.07	-0.96 ± 0.76
